# ALYREF m5C RNA methylation reader predicts bladder cancer prognosis by regulating the tumor immune microenvironment

**DOI:** 10.1097/MD.0000000000037590

**Published:** 2024-04-05

**Authors:** Wengu Pan, Xiaoli Liu, Shuangde Liu

**Affiliations:** a Kidney Transplantation of The Second Hospital, Cheeloo College of Medicine, Shandong University, Jinan, China; b Department of Kidney Transplantation, Multidisciplinary Innovation Center for Nephrology, The Second Hospital of Shandong University, Jinan, China.

**Keywords:** ALYREF, bladder cancer, m5C RNA methylation, prognosis, tumor immune microenvironment

## Abstract

**Background:**

5-Methylcytidine (m5C) methylation is a recently emerging epigenetic modification that is closely related to tumor proliferation, occurrence, and metastasis. This study aimed to investigate the clinicopathological characteristics and prognostic value of m5C regulators in bladder cancer (BLCA), and their correlation with the tumor immune microenvironment.

**Methods:**

Thirteen m5C RNA methylation regulators were analyzed using RNA-sequencing and corresponding clinical information obtained from the TCGA database. The Cluster Profiler package was used to analyze the gene ontology function of potential targets and enriched the Kyoto Encyclopedia of Genes and Genomes pathway. Kaplan–Meier survival analysis was used to compare survival differences using the log-rank test and univariate Cox proportional hazards regression. The correlation between signature prognostic m5C regulators and various immune cells was analyzed. Univariate and multivariate Cox regression analyses identified independence of the ALYREF gene signature.

**Results:**

Nine out of the 13 m5C RNA methylation regulators were differentially expressed in BLCA and normal samples and were co-expressed. These 9 regulators were associated with clinicopathological tumor characteristics, particularly high or low tumor risk, pT or pTNM stage, and migration. Consensus clustering analysis divides the BLCA samples into 4 clusters. Kyoto Encyclopedia of Genes and Genomes (KEGG) pathway enrichment annotation and gene ontology function analysis identified 273 upregulated and 594 downregulated genes in BLCA. Notably, only ALYREF was significantly correlated with OS (*P *< .05). ALYREF exhibited significant infiltration levels in macrophage cells. Therefore, we constructed a nomogram for ALYREF as an independent prognostic factor. Additionally, we observed that both the mRNA and protein levels of ALYREF were upregulated, and immunofluorescence showed that ALYREF was mainly distributed in nuclear speckles. ALYREF overexpression was significantly associated with poor OS.

**Conclusion:**

Our findings demonstrated the potential of ALYREF to predict clinical prognostic risks in BLCA patients and regulate the tumor immune microenvironment. As such, ALYREF may serve as a novel prognostic indicator in BLCA patients.

## 1. Introduction

Bladder cancer (BLCA) is the 10th most common malignancy worldwide, resulting in approximately 170,000 deaths globally every year.^[1]^ It is a highly malignant urogenital tumor characterized by concealed onset and easy misdiagnosis, with both morbidity and mortality increasing in recent years.^[2]^ Distinct proportions of genome subclones result in cellular and molecular heterogeneity in BLCA, which affects both clinical outcomes and therapeutic responses.^[3,4]^ Despite extensive research and considerable progress in diagnosis, clinical interventions, and prognostic assessment for bladder cancer in recent decades, including: diagnostic tools such as artificial intelligence detection,^[5]^ Liquid Biopsy Biomarkers in Urine,^[6]^ discovery of noncoding RNA serum circulating biomarkers,^[7,8]^ circulating tumor marker assays,^[9]^ clinical interventions such as immune checkpoint inhibitors,^[10]^ and prognostic assessment such as systemic combining inflammatory score^[11]^ and Modified Glasgow Prognostic Score,^[12]^ the 5-year survival rate remains low.^[13]^ Consequently, developing a new noninvasive technology to aid clinicians in decision-making is critical.

5-Methylcytosine (m5C) is a widespread mRNA modification discovered in 1925 and is located in the untranslated regions of mRNA transcripts.^[14]^ M5C plays a crucial regulatory role in gene expression, including RNA export, ribosome assembly, and translation.^[15]^ M5C is involved in mRNA, tRNA, rRNA, and ncRNA functions and is associated with RNA stability and translation efficiency.^[16]^ Currently, 13 regulatory factors are known to be involved in m5C methylation. The dynamic modification of m5C is regulated by writers (methyltransferases), readers (binding proteins), and erasers (demethylases).^[17]^ “Writer” complexes, such as NOP2, NSUN2, NSUN3, NSUN4, NSUN5, NSUN6, NSUN7, DNMT1, DNMT3A, DNMT3B, and TRDMT1, increase RNA C5 site methylation.^[18]^ “Reader” protein ALYREF recognizes and binds to methylated RNA, while “Eraser” protein TET2 alters m5C modification through demethylation.^[19]^ Dysregulation of RNA modifications has been associated with several diseases including leukemia,^[20]^ breast cancer,^[21]^ and prostate cancer.^[22]^

In recent years, a large body of research has demonstrated that the tumor immune microenvironment (TIM) plays a vital role in cancer progression and therapeutic efficacy.^[23]^ The TIM is composed of cancer cells, immune cells, and the extracellular matrix, such as myeloid-derived suppressor cells, tumor-associated macrophages, cancer-associated fibroblasts, and immune cells.^[24]^ These nontumor cells can influence the process of tumorigenesis and transfer, thereby having clinical relevance in diagnosis and prognosis.^[25]^

However, the functions of m5C regulators in BLCA remain largely unknown. In this study, we analyzed m5C regulators in BLCA and normal samples using TCGA database and explored the regulation of m5C in BLCA. We also examined clinicopathological characteristics and differences in survival, as well as their impact on TIM, to provide prognostic significance for BLCA treatment.

## 2. Materials and methods

### 2.1. Data collecting and preprocessing

In our study, we aimed to explore the expression profiles of m5C regulatory genes in BLCA compared to normal samples using RNA-sequencing expression profiles (level 3) and corresponding clinical information obtained from the TCGA database (https://portal.gdc.com). The data were preprocessed and displayed as the mean ± SD, and statistical significance was tested using unpaired *t* tests. We also analyzed the association between BLCA and clinical characteristics using Pearson chi-squared tests or Fisher exact tests, as appropriate.^[26,27]^ Table 1 shows the clinical statistical information of the TCGA dataset.

**Table 1:** The clinical characteristics of BLCA patients. The mRNA data and clinical information of 406 BLCA cases patients were downloaded from the TCGA database.

Bladder cancer	Characteristics	Number
Status	Alive	228
	Dead	178
Age	Mean (SD)	68.1 (10.6)
	Median [MIN,MAX]	69 [34,90]
Gender	Female	106
	Male	300
Race	Asian	44
	Black	23
	White	322
pT stage	T1	11
	T2	190
	T3	157
	T4	42
pN stage	NO	236
	NX	164
pM stage	M0	195
	M1	208
pTNM stage	I	2
	II	129
	III	140
	IV	133
Grade	High grade	382
	Low grade	21
New tumor event type	Metastasis	87
	Primary	7
	Recurrence	46
Smoking	Nonsmoking	109
	Smoking	284
Therapy type	Nonradiation	264
	Radiation	10
	Neoadjuvant	10
	No neoadjuvant	396
	Ancillary + Chemotherapy	2
	Chemotherapy	104
	Chemotherapy + Immunotherapy	3
	Immunotherapy	4

### 2.2. Identification of differentially expressed m5C regulatory genes

Thirteen m5C regulatory factors were extracted from previous studies^[28]^ and are shown in Table 2. We then identified differentially expressed m5C RNA methylation regulators between the tumor and normal groups using the rank-sum test. Genes with adjusted *P* values of <.05 were considered as differentially expressed genes (DEGs). We also analyzed the co-expression of m5C regulatory genes in TCGA dataset and identified correlations between m5C regulators and tumor clinicopathological parameters.

**Table 2:** The list of the RNA modifying proteins involve in m5C. The 13 m5C regulatory factors were extracted from the prior studies.

The m5C regulators	Type
NOP2	Writers
NSUN2	Writers
NSUN3	Writers
NSUN4	Writers
NSIN5	Writers
NSUN6	Writers
NSUN7	Writers
DNMT1	Writers
DNMT3A	Writers
DNMT3B	Writers
TRDMT1	Writers
ALYREF	Readers
TET2	Erasers

To perform these analyses, we obtained the current-release (V8) GTEx datasets from the GTEx data portal website (https://www.gtexportal.org/home/datasets) and performed statistical analyses using R software v4.0.3 (R Foundation for Statistical Computing, Vienna, Austria). Statistical significance was set at *P* < .05.^[29,30]^

### 2.3. Exploration of m5C regulatory gene clusters in BLCA

We performed consensus unsupervised clustering analysis based on the expression of m5C regulatory genes to classify patients with BLCA into distinct gene clusters. To verify the stability of the classification, we conducted a BLCA based on the expression of m5C regulatory genes. Additionally, we investigated the relationship between m5C prognostic model, m5C regulatory gene clusters, and clinicopathological features.

Consistency analysis was performed using the Consensus Cluster Plus R package (v1.54.0), with the maximum number of clusters set to 6% and 80% of the total sample drawn 100 times. We used clusterAlg = “hc” and innerLinkage = “ward.D2.” For clustering heatmaps, we used the R software package pheatmap (v1.0.12). The gene expression heatmap retained genes with SD > 0.1, and if the number of input genes was more than 1000, we extracted the top 25% of the genes after sorting by SD. All analysis methods and R packages were implemented in R version 4.0.3.^[31–35]^

### 2.4. Screening DEGs between m5C subtypes and functional analysis

We observed the differential expression of m5C regulatory genes in BLCA and normal samples to obtain clues about their gene function. We used the limma package in R to study the differentially expressed mRNA, defining “Adjusted *P* < .05 and Log2 (Fold Change) > 1 or Log2 (Fold Change) < −1” as the threshold for the differential expression of mRNAs. To further confirm the underlying function of potential targets, we analyzed the data using functional enrichment.

Gene ontology (GO) is a widely used tool for annotating genes with functions, especially molecular functions, biological pathways, and cellular components. Kyoto Encyclopedia of Genes and Genomes (KEGG) Enrichment Analysis is a practical resource for studying gene functions and associated high-level genomic functional information. To better understand the carcinogenesis of mRNA, we employed the Cluster Profiler package (version:3.18.0) in R to analyze the GO functions of potential targets and enrich the KEGG pathway. We used the R software ggplot2 package to draw boxplots and the R software pheatmap package to draw heatmaps.^[36]^

### 2.5. Identification of m5C RNA methylation regulators for predicting the prognosis

We screened the influence of differential genes on the prognosis of BLCA by analyzing the differences in prognosis of different groups using the median value of gene expression as the grouping method according to the expression level. We obtained raw counts of RNA-sequencing data (level 3) and corresponding clinical information from BLCA from TCGA, complying with the guidelines and policies for acquisition and application.

To compare the survival difference between the 2 groups using KM curves, we used Kaplan–Meier (KM) survival analysis and log-rank test. For KM curves, *P* values and hazard ratios (HR) with 95% confidence intervals (CI) were generated using log-rank tests and univariate Cox proportional hazards regression. All analytical methods and R packages were performed using R software version v4.0.3 (The R Foundation for Statistical Computing, 2020). Statistical significance was set at *P* < .05.^[35,37]^

### 2.6. Relationship between m5C regulators and TIM in BLCA

We analyzed the correlation between the risk scores of signature prognostic m5C regulators and various immune cells using TIMER, EPIC, QUANTISEQ, and MCPCOUNTER. We downloaded RNA-sequencing expression (level 3) profiles and corresponding clinical information for BLCA from TCGA.

We used the R software ggstatsplot package to draw correlations between gene expression and immune score and the R software pheatmap package to draw multi-gene correlations. Spearman correlation analysis was used to describe the correlation between quantitative variables without a normal distribution. We considered *P* < .05 statistically significant (at *P* < .05). All analysis methods and R packages were implemented using R version.^[30,31,38–42]^

### 2.7. Construction and evaluation of a nomogram

To assess the independence of the ALYREF gene signature in clinical parameters, we performed univariate and multivariate Cox regression analyses to determine the related HR, 95% CI of HR, and *P* value in the clinical information of the TCGA dataset. We also studied the influence of genes on clinical factors associated with prognosis.

Based on the results of the multivariate Cox regression analysis, we extracted variables with significant differences in prognosis to construct a nomogram that can provide guidance for clinical prognosis. Univariate and multivariate Cox regression analyses were performed to identify appropriate terms for building the nomogram. The *P* value, HR, and 95% CI of each variable are shown in a forest plot using the “forestplot” R package.

We developed a nomogram based on the results of multivariate Cox proportional hazards analysis to predict the X-year overall recurrence. The nomogram provided a graphical representation of the factors that can be used to calculate the risk of recurrence for an individual patient based on the points associated with each risk factor using the “rms” R package.^[43–45]^

### 2.8. Immunohistochemistry, immunofluorescence, and gene expression profiling

We confirmed the expression of ALYREF in BLCA and normal samples using TCGA-GTEx projects and the online GEPIA database (http://gepia.cancer-pku.cn/index.html). We also performed overall survival (OS) and disease-free survival (DFS) analyses for high and low expression of ALYREF in patients with BLCA.

Furthermore, we obtained immunohistochemistry images of ALYREF proteins in BLCA and normal specimens from the Human Protein Atlas (https://www.proteinatlas.org/), where we downloaded immunofluorescence images.^[46]^

### 2.9. Validation of the gene signature

We calculated the risk score of each sample based on the signature gene and generated the risk score distribution of the sample using GEPIA. Additionally, we used the “time ROC” package to perform a receiver operating characteristic (ROC) analysis to explore the prediction accuracy of survival rates.

GEPIA allows for analysis of overall survival (OS) or DFS (also called relapse-free survival and RFS) based on gene expression. The platform uses the log-rank test, also known as the Mantel–Cox test, for hypothesis testing. Cohort thresholds can be adjusted and gene pairs can be used. The Cox proportional hazard ratio and 95% confidence interval information were also included in the survival plot. The platform also allows the search for genes that are most associated with patient survival.

## 3. Results

### 3.1. Different expression of m5C RNA methylation regulators in BLCA

We downloaded the corresponding clinical information of 406 BLCA and 40 normal samples from TCGA data portal (Table 1), which included alive or dead status, age, sex, race, pT stage, pN stage, pM stage, pTNM stage, high or low grade, new tumor event type, smoking status, and therapy type. Additionally, we identified 13 m5C RNA methylation regulators, including 11 writers (NOP2, NSUN2, NSUN3, NSUN4, NSUN5, NSUN6, NSUN7, DNMT1, DNMT3A, DNMT3B, and TRDMT1), 1 reader (ALYREF), and 1 eraser (TET2), as shown in Table 2.

**Figure 1.**
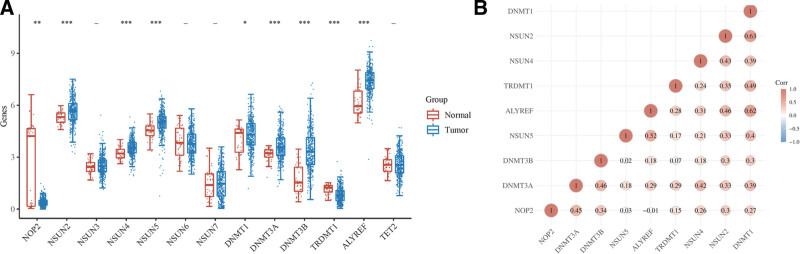
The expression of m5C genes and its correlations in BLCA and normal samples. In (A), the abscissa represents m5C genes, and the ordinate represents the expression distribution of gene. The statistical difference of 2 groups was compared through the Wilcox test. In (B), a heatmap shows the correlation in 9 m5C genes. The abscissa and ordinate represent genes, different colors represent different correlation coefficients (blue represents positive correlation whereas red represents negative correlation), the darker the color, the stronger the relation. *P < .05, **P < .01, ***P < .001 stand for significance levels.

We studied the relationship between m5C RNA methylation regulators and BLCA by analyzing the expression of 13 m5C RNA methylation regulators in BLCA samples compared with normal tissue samples. TCGA data showed that 9 m5C RNA methylation regulators were abnormally expressed in BLCA (Fig. 1A). In the BLCA tissues, NOP2 (*P* < .01), NSUN2 (*P* < .001), NSUN4 (*P* < .001), NSUN5 (*P* < .001), DNMT1 (*P* < .05), DNMT3A (*P* < .001), DNMT3B (*P* < .001), TRDMT1 (*P* < .001), and ALYREF (*P* < .001) showed significant differences in expression. NOP2 and TRDMT1 expression levels were significantly lower in BLCA tissues than in normal tissues. NSUN2, NSUN4, NSUN5, TRDMT1, DNMT3A, DNMT3B, and ALYREF were significantly overexpressed in BLCA tissues. Co-expression analysis revealed varying degrees of correlation among the 9 m5C regulatory genes (Fig. 1B).

**Figure 2.**
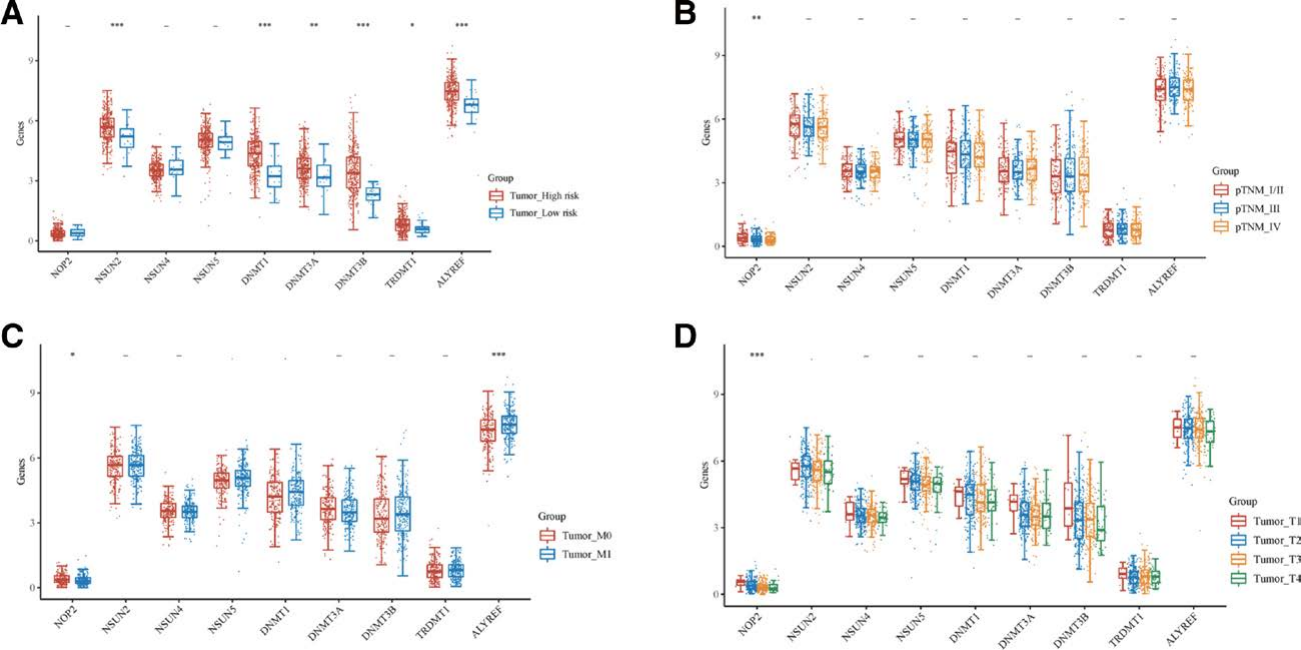
The expression of 9 m5C genes in different clinicopathology characteristic of BLCA. The abscissa represents the genes, and the ordinate represents the expression distribution of these genes. The statistical difference of 2 groups was compared through the Wilcox test, significance difference of 3 groups was tested with Kruskal–Wallis test. Different colors represent different groups, top represents the significance *P* value, *P < .05, **P < .01, ***P < .001.

To investigate the clinical relationship between the 9 differentially expressed m5C RNA methylation regulators in BLCA, we analyzed their expression levels in BLCA samples from TCGA database in relation to different clinicopathological characteristics. Our results showed that NSUN2, DNMT1, DNMT3A, DNMT3B, TRDMT, and ALYREF were related to high or low tumor risk, while NOP2 was related to the pTNM stage of tumors. In addition, both NOP2 and ALYREF were associated with tumor migration, and NOP2 was related to the pT stage of the tumors. (Fig. 2).

#### 3.1.1. Screening DEGs in m5C subtypes and functional analysis.

**Figure 3.**
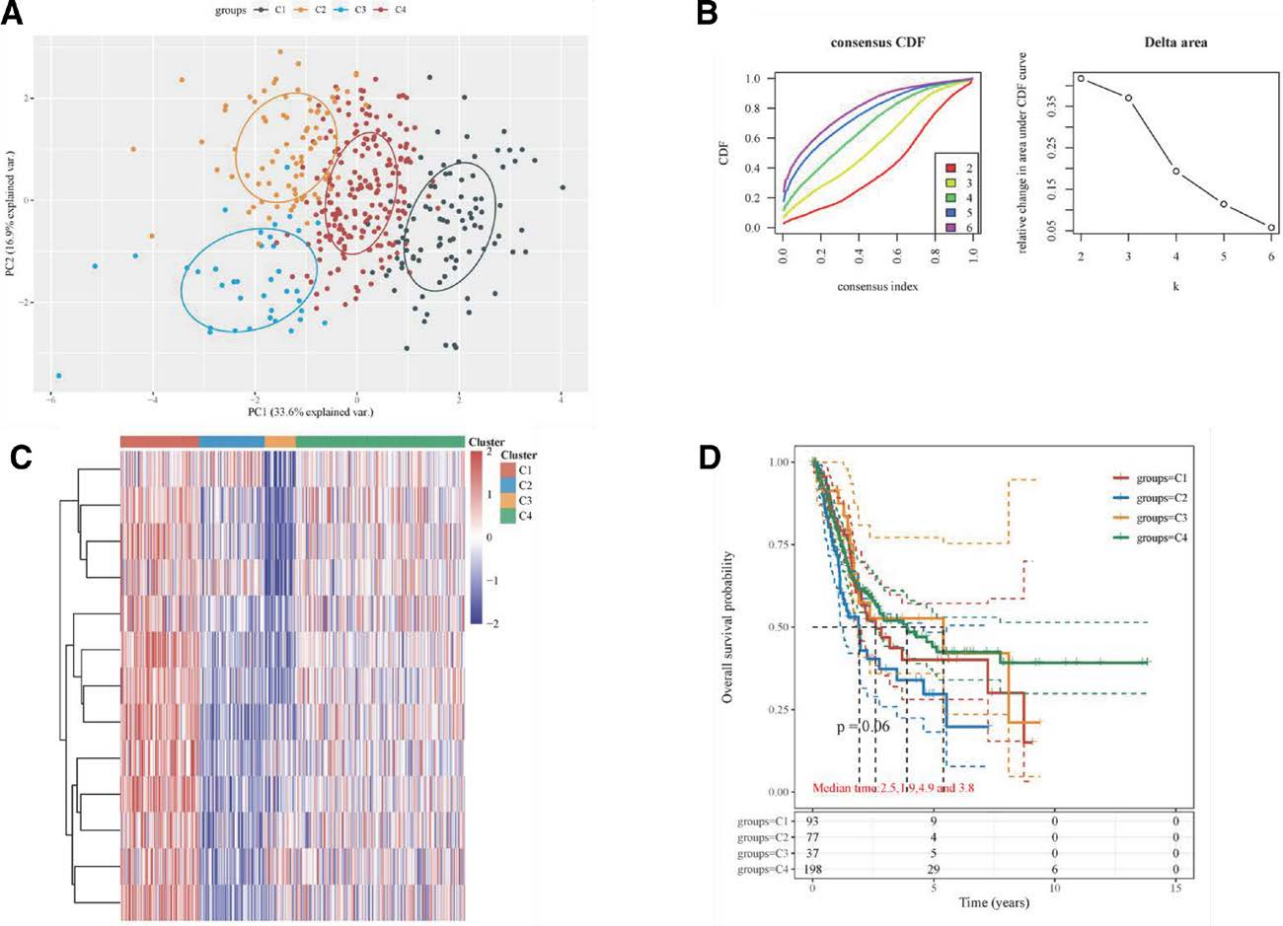
Consensus clustering cumulative distribution function (CDF) and relative change in the area under the CDF curve. The relative change in area under the CDF curves when cluster number varying from *k −* 1 to 6. The abscissa represents category number 4, and the ordinate represents the relative change in the area. Consistency of clustering results heatmap (*k* = 4), Rows and columns represent samples, and the different colors represent different types. And the expression heatmap of 9 m5C genes in different subgroups, red represents high expression, and blue represents low expression. Kaplan–Meier survival analysis of the different groups was from TCGA by log-rank test. HR (95%Cl), the median survival time (LT50) for different groups.

Based on the similarity in the expression of the 9 m5C RNA methylation regulators, dividing the BLCA samples into 4 clusters (*k* = 2; Fig. 3A–D) was the optimal approach in the consensus cluster analysis. Additionally, the OS rates in clusters 1 to 4 were significantly different, indicating that these 9 regulators could be used for the prognostic classification of BLCA.

**Figure 4.**
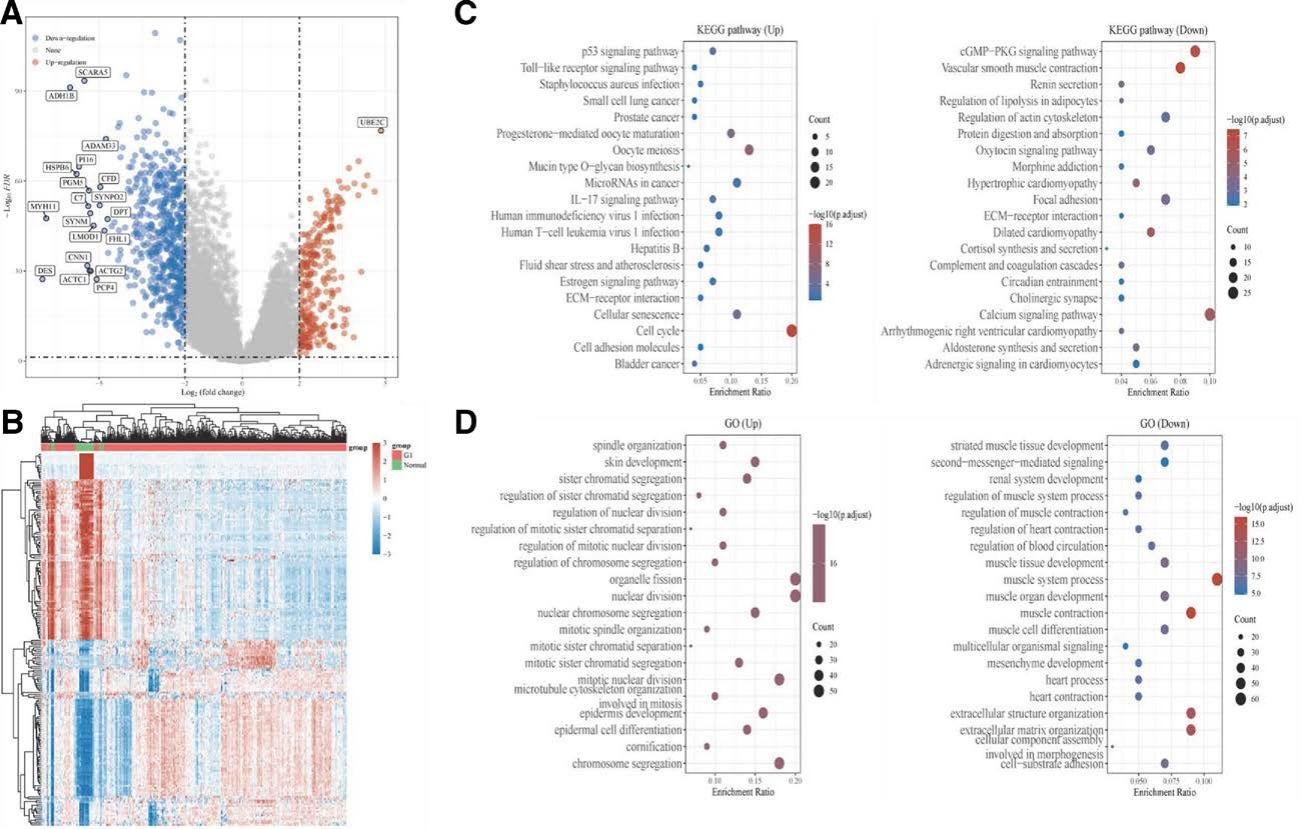
Screening differentially expressed genes between m5C subtypes and functional analysis. (A) In Volcano plot, the volcano plot was constructed using the fold change values and *P*-adjust. Red dots indicate upregulated genes; blue dots indicate downregulated genes; gray dots indicate not significant in (A). (B) The heatmap of the differential gene expression, different colors represent the trend of gene expression in different tissues in (B). (C) The top 50 upregulated genes and top 50 downregulated genes were showed in this figure. The enriched Kyoto Encyclopedia of Genes and Genomes (KEGG) signaling pathways were selected to demonstrate the primary biological actions of major potential mRNA. The abscissa indicates gene ratio and the enriched pathways were presented in the ordinate in (C). (D) Gene ontology analysis of potential targets of mRNAs, including the biological process, cellular component, and molecular function of potential targets in (D). Colors represent the significance of differential enrichment, the size of the circles represents the number of genes, the larger the circle, the greater the number of genes. In the enrichment result, *P* < .05 or FDR < 0.05 is considered to be a meaningful pathway (enrichment score with −log10 (*P*) of more than 1.3).

To further analyze the DEGs between m5C subtypes and their functional implications, we identified 273 upregulated and 594 downregulated genes in BLCA samples compared to those in normal samples. Representative DEGs are depicted in the volcanic map (Fig. 4A) and a heat map of the 100 most significantly changed genes is presented in Fig. 4B. We performed KEGG pathway enrichment and GO function analyses of the upregulated and downregulated DEGs.

The top 10 pathways annotated for upregulated DEGs in KEGG pathway enrichment analysis are presented in Fig. 4C, including the p53 signaling pathway, Toll-like receptor signaling pathway, microRNAs in cancer, IL-17 signaling pathway, estrogen signaling pathway, ECM-receptor interaction, cellular senescence, cell cycle, cell adhesion molecules, cGMP-PKG signaling pathway, and pathways in bladder cancer. Additionally, we analyzed the GO functions of upregulated and downregulated DEGs and presented the top 20 annotations in Fig. 4D.

### 3.2. ALYREF as a prognostic factor in BLCA

**Figure 5.**
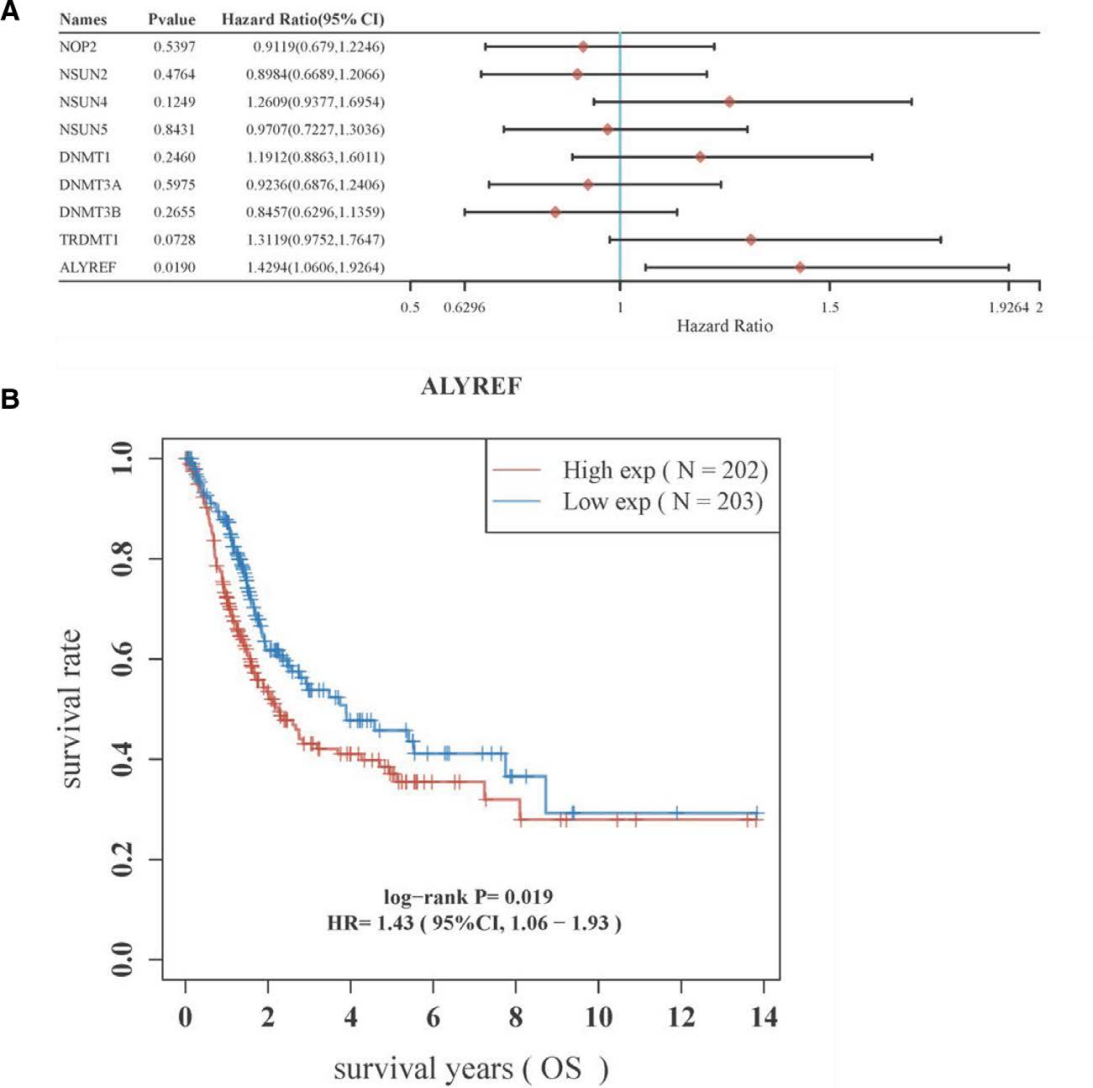
Kaplan–Meier survival analysis of the gene signature from TCGA database. Construct of m5C RNA methylation regulator signature and assess of its prognostic value. (A) The coefficients of ALYREF. (B) Survival analysis of the high and low-risk groups of TCGA PC cohort.

We conducted KM survival analysis to assess the association between the signature of the 9 m5C RNA methylation regulators and OS using log-rank tests and univariate Cox proportional hazards regression. Our analysis revealed that only ALYREF expression significantly correlated with OS (*P* < .05; Fig. 5A). We also analyzed BLCA patients in the high-risk and low-risk groups using KM survival analysis and found that the low-risk group had a significantly improved OS compared to the high-risk group (Fig. 5B; *P* = .019).

### 3.3. Immune infiltration analysis of ALYREF and its nomogram

**Figure 6.**
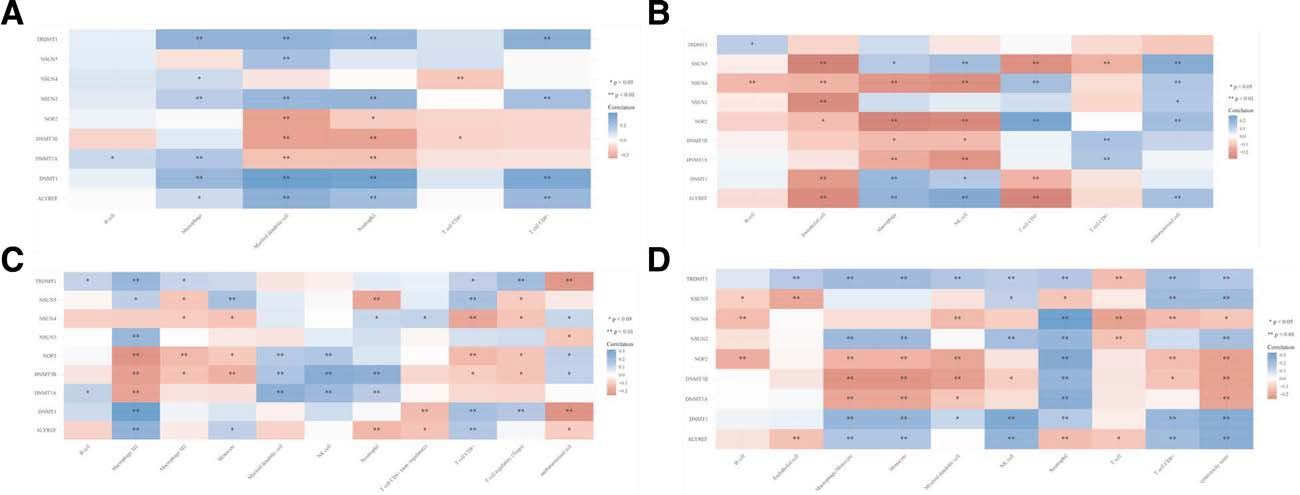
The correlations between gene expression and immune score. Tumor immune microenvironment (TIM) relationships were analyzed with Spearman by TIMER (A), EPIC (B), QUANTISEQ (C), and MCPCOUNTER (D). The abscissa represents the different TIM cells, and the ordinate represents the m5C genes. Different colors represent different correlation coefficients (blue represents positive correlation and red represents negative correlation), the darker the color, the stronger the relation. **for *P* < .01, *for *P* < .05.

We analyzed the expression levels of ALYREF in tumor microenvironment-related cells using TIMER, EPIC, QUANTISEQ, and MCPCOUNTER databases. These databases include immune, inflammatory, and stromal cells that play a crucial role in tumorigenesis, development, metastasis, recurrence, and drug resistance. The results showed that ALYREF had a significant infiltration level of macrophage cells in all 4 databases, as shown in Fig. 6.

**Figure 7.**
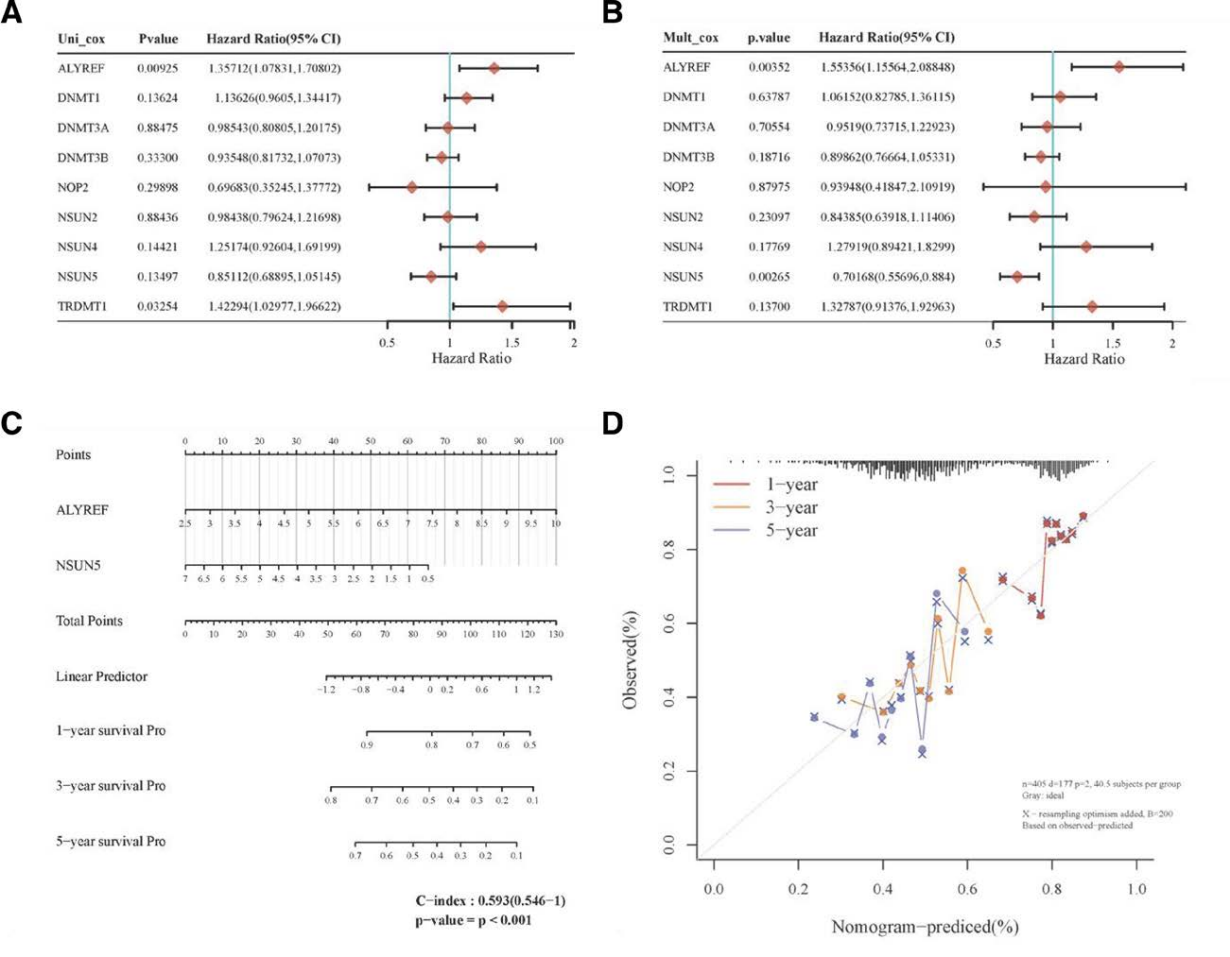
The identification of prognostic factor for OS and the development of the nomogram. (A) Univariate Cox analysis. (B) Multivariate Cox analysis. (C) Nomogram for OS in BLCA patients. (D) The calibration curves for 1 y, 3 y, and 5 y. The *P* value, risk coefficient (HR) and confidence interval are analyzed by univariate and multivariate Cox regression. Nomogram can predict the 1 y, 3 y, and 5 y overall survival of BLCA patients. Calibration curve was for the overall survival nomogram model in the discovery group. The dashed diagonal line represents the ideal nomogram, and the blue line, red line, and orange line represent the 1 y, 3 y, and 5 y of the observed nomogram.

To evaluate the independence of the m5C regulator signature with clinical parameters, we used univariate and multivariate Cox regression to analyze the HR, 95% CI of HR, and *P* value in the clinical information of the TCGA dataset. Our analysis revealed that the ALYREF signature had a good predictive performance and clinical application value. We constructed a nomogram in Fig. 7 based on the results of the univariate and multivariate Cox regression analyses.

We found that the *P* value of ALYREF was <.05 in both univariate and multivariate Cox regression analyses, indicating that ALYREF is an independent indicator for predicting the prognosis of BLCA. The calibration curves for the prediction of 1, 3, and 5 years survival rates were close to the standard curves, indicating that the signature had a good prediction performance.

### 3.4. Upregulation of ALYREF with poor clinical outcomes of BLCA

**Figure 8.**
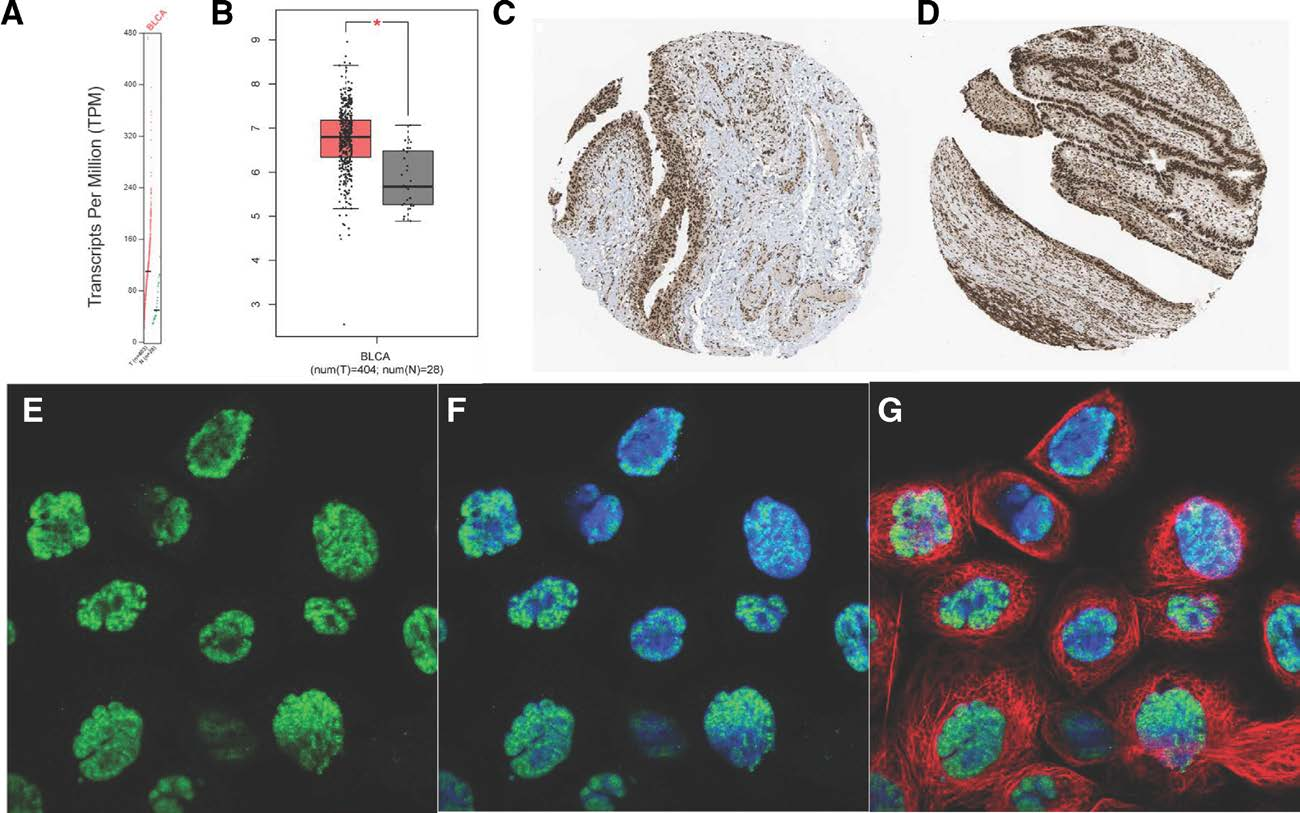
*ALYREF* was upregulated in BLCA. Upregulation of *ALYREF* mRNA in BLCA specimens. The picture shows the mRNA expression levels from the GEPIA (A, B). The immunohistochemistry (C, D) and immunofluorescence (E–G) images were from the Human Protein Atlas. The nucleus was stained blue, the microtubules were stained red, and the *ALYREF* proteins were stained green. *P < .05.

We observed that ALYREF was upregulated at both the mRNA and protein levels in BLCA. The mRNA expression levels from the GEPIA database showed that ALYREF was upregulated at the mRNA level in BLCA cells. Immunohistochemistry and immunofluorescence images from The Human Protein Atlas database also demonstrated that ALYREF was upregulated at the BLCA protein level. Immunofluorescence results further showed that ALYREF was mainly distributed in nuclear speckles (Fig. 8).

**Figure 9.**
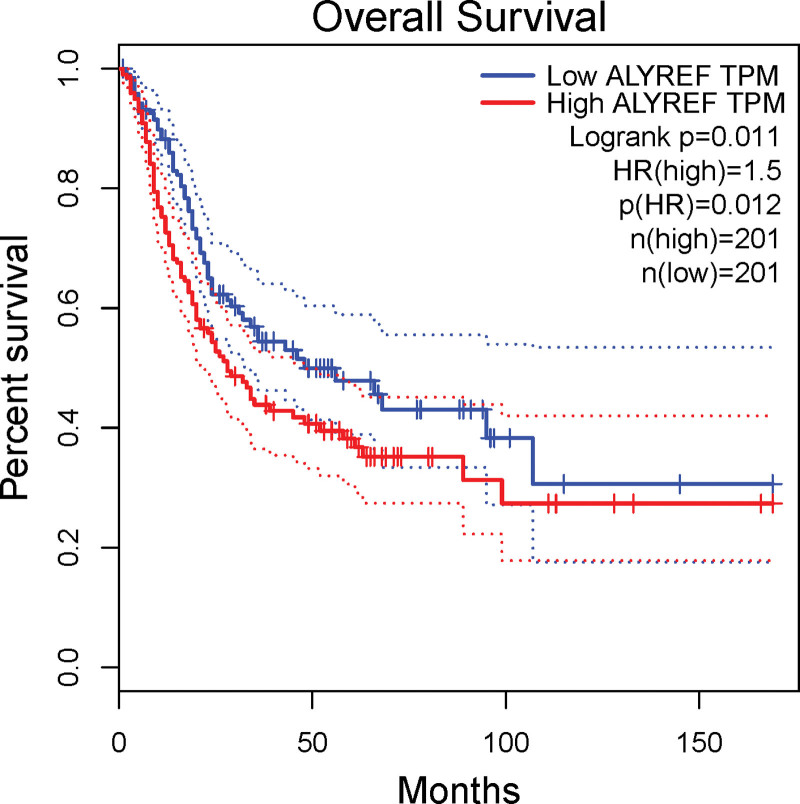
*ALYREF* was associated with poor clinical outcomes. *ALYREF* was upregulated in BLCA and associated with poor clinical outcomes. Kaplan–Meier curves for *ALYREF.*

We also found that ALYREF expression was significantly associated with the prognosis of patients with BLCA. Patients with high ALYREF expression had a shorter DFS (*P* = .011; Fig. 9). Furthermore, high ALYREF expression was associated with poorer OS. These findings suggest that ALYREF plays a crucial role in the development and progression of BLCA and could serve as a potential prognostic biomarker for BLCA (Fig. 9).

## 4. Discussions

As the 9th most frequently diagnosed cancer worldwide, the incidence of BLCA has been increasing year by year, and there are gender differences in incidence and prognosis.^[47,48]^ Although with the upgrading of clinical prediction tools for bladder cancer,^[7,49]^ the continuous development of therapeutic tools such as surgery, chemotherapy, and radiotherapy,^[50]^ as well as the improvement of prognostic assessment systems,^[12,51]^ these advances have effectively prolonged the survival of bladder cancer, but the complexity of clinical complications and associated risk factors^[52–54]^ have led to an unsatisfactory prognosis.^[55]^ Because of the incomplete understanding of the pathological basis of bladder cancer, it is an important matter to study in depth the genetic mechanisms and epigenetic factors involved in bladder cancer in order to identify novel clinical therapeutic targets and diagnostic biomarkers.

Posttranscriptional modifications of mRNA, including 5-terminal capping, pre-mRNA splicing, polyadenylation, and mRNA export, are regulated by a complex network of writers, erasers, and readers.^[56,57]^ These proteins work together to dynamically regulate RNA modifications. The internal modifications of mRNA include N6-methyladenosine (m6A), N1-methyladenosine (m1A), 5-methylcytosine (m5C), and N7-methylguanosine (m7G).^[58]^

In recent years, a large number of studies have shown that RNA modifications (acetylation, m6A) affect the progression and prognosis of bladder cancer.^[59–62]^ For example, NAT10 is closely related to the tumorigenicity of bladder cancer, and its overexpression mediates the acetylation modification of mRNA, such as BCL9L, SOX4, and AKT1, in patients with BLCA, thus affecting the progression of bladder cancer.^[59]^ High expression of YTHDF2 is a risk factor for poor prognosis of patients with BLCA. YTHDF2 promotes bladder cancer cell proliferation and tumor growth, and RIG-I is a downstream target of YTHDF2. YTHDF2 can regulate m6A modification to induce degradation of DDX58 mRNA encoding RIG-I, and thus inhibit apoptosis and promote cell proliferation in BLCA cells.^[60]^ This suggests that the m6A/acetylation regulator plays an important role in BLCA progression. However, relatively few studies have been conducted on the role of m5c regulatory genes in BLCA. The present study is the first systematic study to investigate the expression and prognostic model of 13 m5C RNA methylation regulators in BLCA progression, and to investigate the enrichment pathway, TIM correlation, OS, clinicopathological features, and risk scores of the clinical samples among the subgroups of m5C-regulated genes. The results of this study indicated that m5C regulated genes play an important role in bladder cancer progression and prognosis. In addition, we identified a key regulator, ALYREF, which is upregulated in BLCA and associated with poor prognosis.

To establish a risk signature-related m5C RNA methylation regulator, we collected information on 406 BLCA and 40 normal samples from the TCGA database. We then analyzed the expression of 13 reported m5C RNA methylation regulators in BLCA and normal samples based on published papers. We used log-rank tests and univariate Cox proportional hazards regression to evaluate the relationship between the 9 methylation regulators and OS. We found that only ALYREF expression significantly correlated with OS. Aly/REF export factor (ALYREF, also known as THOC4) functions as a specific mRNA m5C-binding protein that regulates mRNA export.^[19]^ ALYREF was upregulated at the mRNA and protein levels, and immunofluorescence results demonstrated that it was mainly distributed in the nuclear speckles.

Our study revealed the physical and functional interactions between the immune-related gene signature and expression of m5C regulators in tumor microenvironment-related cells. We found that the expression levels of ALYREF in the tumor microenvironment-related cells of BLCA were distinctly correlated with the TIM, which is why the other 8 different m5C regulators were not related to prognosis. TIM has also been shown to play an important role in cancer development and progression and has a significant impact on the prognosis of cancer patients, and immune subtypes of tumors play a very important role in developing therapeutic strategies and evaluating patient prognosis. Several studies have elucidated the correlation between TIM and RNA modification (m5C).^[63,64]^ Using the results of univariate and multivariate Cox regression analyses, we constructed a nomogram with ALYREF as the only significant factor that correlated with OS. This predictive model accurately predicts the prognostic status of BLCA.

Dynamic, tissue-specific, conserved m5C modification of mRNAs is mainly written by NOP2/Sun RNA methyltransferase family member 2 (NSUN2) and specifically read by the Aly/REF export factor (ALYREF, also named THOC4) and Y-box binding protein 1 (YBX1). ALYREF specifically reads m5C regions through lysine residue K171. Previous studies have reported that overexpression of ALYREF promotes bladder cancer cell proliferation via PKM2-mediated glycolysis. Furthermore, high PKM2 and ALYREF expression predicts poor survival in patients with bladder cancer.^[65]^

ALYREF has been found to stabilize PKM2 mRNA through an m5C-mediated pathway, promoting glycolysis and proliferation of BLCA cells, making it a tumor-driver gene in BLCA. ALYREF exerts a carcinogenic effect in BLCA by upregulating PKM2 expression, which mediates its effect in BLCA cells. HIF-1α can upregulate ALYREF expression in BLCA cells, indirectly activating PKM2 expression and directly activating its transcription.

## 5. Clinical implications

Our study suggests that m5C may play a critical role in the hypoxia-glycolysis network, and that the HIF-1α/ALYREF/PKM2 signaling pathway may be a potential therapeutic target for BLCA. As high expression of PKM2 and ALYREF is associated with poor outcomes in patients with BLCA, they may provide promising biomarkers to guide early diagnosis and treatment.^[65]^

## 6. Study limitations

This study had some limitations. First, this study is an analysis using public databases and lacks validation of our own cohort; therefore, we will further investigate the m5C-related regulators in our own BLCA cohort. Second, the targeted downstream pathways of ALYREF were not further explored, which may cause some bias in estimating the targeted drugs and should be further investigated. Third, the number of patients is still small for selecting the appropriate model, and the results still need further validation. Finally, there are fewer studies related to other highly expressed biomarkers in this study. The role of these biomarkers on BLCA needs to be further explored with the improvement of technical means.

## 7. Conclusions

In conclusion, our research showed significant differences in the expression of m5C regulators in BLCA and adjacent tissues. We have developed prognostic risk markers for the m5C regulator, ALYREF, and its effects on TIM make it a potential prognostic marker in BLCA and provide strategies for the treatment of this disease.

## Author contributions

**Conceptualization:** Wengu Pan, Shuangde Liu.

**Data curation:** Wengu Pan, Shuangde Liu.

**Formal analysis:** Wengu Pan, Shuangde Liu.

**Funding acquisition:** Wengu Pan, Shuangde Liu.

**Investigation:** Wengu Pan, Shuangde Liu.

**Methodology:** Wengu Pan, Shuangde Liu.

**Project administration:** Xiaoli Liu, Shuangde Liu.

**Resources:** Xiaoli Liu, Shuangde Liu.

**Software:** Xiaoli Liu, Shuangde Liu.

**Supervision:** Xiaoli Liu, Shuangde Liu.

**Validation:** Xiaoli Liu, Shuangde Liu.

**Visualization:** Xiaoli Liu, Shuangde Liu.

**Writing—original draft:** Xiaoli Liu, Shuangde Liu.

**Writing—review & editing:** Xiaoli Liu, Shuangde Liu.
